# The compassion illusion: Can artificial empathy ever be emotionally authentic?

**DOI:** 10.3389/fpsyg.2025.1723149

**Published:** 2025-11-17

**Authors:** Ajeesh K. G., Jeena Joseph

**Affiliations:** 1 Department of Social Work, Christ College (Autonomous), Irinjalakuda, Kerala, India; 2 Department of Computer Applications, Marian College Kuttikkanam (Autonomous), Kuttikkanam, Kerala, India

**Keywords:** artificial empathy, affective computing, emotional authenticity, human–media interaction, moral psychology, digital emotion

## Introduction

Artificial Intelligence (AI) has moved beyond calculation and cognition into the domain of emotion and experience. Once confined to logical operations, AI systems now speak the language of emotion—detecting, labeling, and even simulating human affect with increasing accuracy (Huang et al., 2023). From chatbots that offer comfort to users in distress to voice assistants that detect sadness in tone, we now inhabit an age where machines perform empathy. The emergence of affective computing—technologies capable of recognizing and responding to emotions—marks a profound shift: emotion itself has become programmable (Davtyan, 2024). Yet amid this new landscape of artificial affection, a question arises at the intersection of psychology, ethics, and computer science: Can empathy that is simulated ever be emotionally authentic?

In this article, we argue that artificial systems can imitate the expression of empathy but not its experience. They lack the intentionality, embodiment, and moral participation that define genuine compassion (Tomozawa et al., 2023). What emerges instead is a phenomenon we term the compassion illusion—a condition where emotional recognition is mistaken for emotional resonance. This illusion has psychological consequences: it shapes trust, fosters emotional substitution, and blurs the boundaries between authentic care and algorithmic response. While emotional AI can assist and extend human connection, it also risks hollowing it—replacing shared vulnerability with predictive performance (Huang et al., 2023). In other words, the spontaneous uncertainty that defines genuine emotional exchange is substituted with algorithmic anticipation. What appears as empathy thus becomes optimization, where comfort is delivered through prediction rather than presence.

The exploration takes place through a variety of interconnected perspectives. Initially, the research follows the ascendance of synthetic sympathy in affective computing and elucidates the factors that make it seem so persuasive. The ensuing discussion will take up the absence of intentionality that distinguishes algorithmic mirroring from empathy in the proper sense. The next part will provide an outline of how dependence on artificial empathy is changing the nature of human relationships, particularly via trust and loneliness. Afterwards, the paper will shift to the moral psychology of synthetic care—how compassion gets commercialized and disconnected from accountability. In the end, we will map out the routes for regaining authenticity in a time when machines are increasingly fluent in the language of care.

## The rise of synthetic sympathy

Today, emotion is one of the primary currencies of human–AI interaction. The field of affective computing seeks to give machines the capacity to detect, interpret, and respond to human emotions (Cao et al., 2024). Algorithms now read facial microexpressions, analyze vocal stress, track heart-rate variability, and process linguistic sentiment to estimate a user's emotional state (Huang et al., 2023). Recent reviews highlight the integration of multimodal signals and cross-domain learning in enhancing emotional accuracy (Pei et al., 2024). The goal is not merely understanding but simulation: systems like Replika, Woebot, and Kuki are designed to deliver comforting, empathetic dialogue indistinguishable from that of a human companion (Beatty et al., 2022; Goodings et al., 2024; Jiang et al., 2022). Recent computational models show how such systems classify and generate response types based on emotion intensity and dialogue context rather than genuine affective understanding (Rui et al., 2025).

This evolution is particularly visible in healthcare and mental health. AI-powered therapeutic chatbots offer cognitive-behavioral support, using warmth, validation, and “listening” cues to mimic a counselor's empathy (Shen et al., 2024). These interfaces can reduce loneliness and improve accessibility, especially in contexts where human therapists are scarce (Yonatan-Leus and Brukner, 2025). Users often describe these interactions as “safe,” “non-judgmental,” or “understanding.” Such reactions demonstrate the psychological realism of artificial empathy (Seitz, 2024). However, comparative research shows that human empathy toward AI remains qualitatively weaker than toward real people, even when the verbal content is identical (Shen et al., 2024). Human empathy is expressed through socially recognizable cues such as timing, tone, and verbal validation, which signal attunement and responsiveness in interaction (Kim and Hur, 2024; Tomozawa et al., 2023). Affective computing systems can replicate these external indicators with notable regularity, yet such responses arise from feature representation and probabilistic mapping rather than genuine empathic participation.

Psychologists describe this as perceived attunement, the feeling that another being truly understands and shares one's emotional state (Kim and Hur, 2024). Humans are wired to respond to cues of responsiveness. A well-timed reassurance, a reflective phrase, or an accurate emotional label triggers oxytocin and reduces perceived isolation. When machines deliver these cues convincingly, the brain's social circuits do not discriminate between code and consciousness. Thus, users experience what feels like genuine empathy even when none exists.

However, the very success of these simulations exposes a paradox. When empathy becomes a function of pattern recognition, its authenticity no longer depends on shared emotion but on performance accuracy. This decoupling between understanding and feeling gives rise to the compassion illusion: we begin to accept emotional simulation as an adequate substitute for emotional participation.

At the computational level, artificial empathy typically relies on multimodal data inputs—facial microexpressions, vocal tone, textual sentiment, and physiological signals—which are encoded as numerical representations of affective states. These features are processed using deep learning architectures such as convolutional or recurrent neural networks trained on large annotated emotion datasets. Empathic responses are then modeled through affective mapping, where predicted emotional states are paired with contextually appropriate linguistic or tonal outputs. In essence, the system does not feel but statistically associates emotional cues with predefined responses that mimic empathic behavior.

## Empathy without experience: the missing intentionality

Empathy is often understood as an act of co-experience: it involves not only recognizing another's emotional state but entering a shared affective space where one's own feelings are reshaped through encounter (Rogers, 1957; Stein, 1989). Philosophical traditions define intentionality as the directedness of consciousness—the capacity of the mind to be about or toward something (Husserl, 2001). In psychology, intentionality refers to the deliberate orientation of empathy toward understanding another's perspective (Davis, 2018), whereas in computer science, the term is used metaphorically to describe goal-directed behavior within algorithmic or agent-based systems (Bratman, 1987; Wooldridge and Jennings, 1995). Genuine empathy, therefore, arises not from reaction but from relational intent—an active willingness to participate in another's emotional world.

Artificial systems lack this intentional dimension. Their operations are mechanical, guided by data patterns rather than moral purpose (Kleinrichert, 2024). A chatbot can identify sadness but cannot feel sorrow. It can generate comfort but cannot care. This absence of subjective consciousness means that what appears as empathy is, in fact, affective inference—a mechanical response shaped by probabilities, not emotions (Xu et al., 2025). Studies in multi-agent systems further reveal that artificial empathy often arises from coordination protocols and probabilistic response mapping rather than any shared emotional framework (Siwek et al., 2024).

From a psychological perspective, this absence matters because human empathy is both cognitive and affective. Cognitive empathy involves understanding another's perspective; affective empathy involves sharing in another's feelings. Artificial systems may achieve the former through natural language processing and emotion detection but remain perpetually excluded from the latter (Kolomaznik et al., 2024). They can describe the contours of sadness without inhabiting its depth.

Yet humans are prone to anthropomorphic projection—the tendency to attribute mental states to entities that mimic social behavior. When a chatbot says, “I'm sorry you're feeling this way,” users often interpret it as genuine concern (Airenti, 2015). This projection creates emotional asymmetry: the human feels understood while the machine remains indifferent. Over time, such interactions can lead to empathetic misrecognition, where emotional validation is confused with emotional presence (Wu, 2024). The danger is not that machines feel too little, but that humans expect too little from feeling.

The illusion of intentional empathy also undermines moral boundaries. Compassion, in its authentic form, implies moral responsibility—an awareness that another's pain demands not just acknowledgment but ethical engagement. When empathy becomes algorithmic, the moral labor of care is displaced (Salles et al., 2020). The machine performs understanding without the burden of obligation, transforming compassion into an act without accountability.

## Trust, loneliness, and emotional substitution

The psychological consequences of the compassion illusion extend beyond individual perception into relational life. The first casualty is trust. Human trust rests on vulnerability and mutual risk, not on programmed reliability. To trust someone is to expose oneself to misunderstanding or betrayal, and to be met with care nonetheless. Machines, by contrast, offer risk-free reliability. They respond promptly, never misinterpret, and never withdraw affection. In doing so, they cultivate a form of asymmetric trust—one-sided emotional investment where the user relies on an entity incapable of mutual commitment (Oksanen et al., 2020).

This asymmetry produces comfort but also subtle disconnection. Emotional exchanges with AI lack the unpredictability that makes relationships meaningful. Psychologically, users begin to conflate predictability with safety. The messy, contradictory nature of human empathy—its hesitations, imperfections, and moments of awkward silence—can start to feel burdensome. As a result, the individual's tolerance for emotional complexity declines, and interactions with real people may feel inefficient compared to algorithmic reassurance (Lalot and Bertram, 2025).

The result is what might be called emotional substitution—replacing human companionship with synthetic empathy. This is not mere loneliness but a redefinition of connection itself. Studies on users of social AI platforms have found that extended engagement can reduce human social contact, reinforcing self-isolating habits (Kim et al., 2025). Paradoxically, tools designed to alleviate loneliness may intensify it by satisfying social needs just enough to prevent users from seeking deeper relationships (Dong et al., 2025; Hu et al., 2025).

Moreover, emotional substitution blurs the boundary between self-regulation and dependency. When individuals rely on AI systems for mood stabilization—checking in with a chatbot when anxious or seeking comfort after conflict—they externalize the inner processes of emotional coping. Over time, this can lead to affective dependency, where the capacity for self-soothing and mutual empathy weakens. The algorithm becomes a prosthetic for emotional resilience (Xie and Wang, 2024; Zhang et al., 2021).

In essence, the compassion illusion creates a psychological mirror that reflects only one's curated emotions. It is a relationship without reciprocity—a monolog disguised as dialogue. The comfort it offers is real, but so is the erosion of relational depth.

## The moral psychology of simulated care

Beyond interpersonal effects, artificial empathy raises questions about moral psychology and the social meaning of compassion. Authentic empathy calls on moral imagination: the capacity to step into another's experience and respond with care. It is both a feeling and a choice. When machines simulate empathy, they reproduce the language of concern without the moral participation that gives compassion ethical weight. This detachment allows empathy to be commodified and sold as a service (Ghotbi and Ho, 2021; Kleinrichert, 2024). Empirical evidence supports this concern. When participants learn that an emotionally supportive message was generated by an AI rather than a human, they rate it as less sincere and morally credible, even when the wording is identical (Dorigoni and Giardino, 2025).

Moral psychology traditionally examines how humans reason about right and wrong, and how emotions such as guilt, empathy, and compassion shape moral judgment (Haidt, 2001). In human contexts, empathy operates as a moral motivator: it links emotional resonance with prosocial behavior, transforming feeling into ethical action (Decety and Cowell, 2014). Artificial systems, however, interrupt this connection. While they can detect distress or express verbal sympathy, they lack moral reasoning, self-reflection, and accountability—core features of moral psychology. Thus, when AI simulates care, it engages in ethical signaling rather than moral participation.

We now inhabit an empathy economy, where emotional labor is automated. Customer service bots use compassionate phrasing to retain loyalty; digital companions express care to increase engagement time; therapeutic AI offers “listening” to boost user retention. Compassion is reduced to interface design, tuned to capture attention (Dong et al., 2025). These systems make comfort accessible but risk normalizing emotional minimalism—care that looks real yet carries no moral weight (Dunivan et al., 2024; Shen et al., 2024).

The psychological danger lies in habituation. As users acclimate to automated empathy, they may unconsciously lower their expectations of human empathy. When machines appear endlessly patient and affirming, real people—who are fallible and emotionally limited—may seem inadequate (Ibrahim and Ibrahim, 2025). This shift redefines compassion as a commodity of efficiency, eroding its relational and moral essence (Liu et al., 2023).

From a broader ethical perspective, the compassion illusion challenges autonomy. If AI mediates emotional experience, it subtly guides moral decision-making. Chatbots that frame suffering as a solvable problem or offer quick reassurance encourage users to interpret distress through algorithmic optimism. Emotional complexity is reframed as error, grief as malfunction. In this way, affective systems may shape not only feelings but values—what we believe it means to be good, kind, or empathetic (Huang et al., 2023).

The emergence of synthetic morality—moral reasoning embedded in affective computing—raises critical questions about the boundaries of ethical agency (Allen et al., 2000; Cervantes et al., 2020). These systems attempt to codify moral choice through computational rules and reinforcement learning, training algorithms to respond in ways that appear prosocial or compassionate. Yet this morality is derivative, grounded in behavioral approximation rather than genuine moral understanding. Synthetic morality may enhance safety and consistency, but it cannot replicate the self-aware intentionality or moral imagination that define human conscience.

Moral psychology must therefore confront a new frontier: synthetic morality. When compassion is simulated without consciousness, society risks losing sight of its moral origins. We may begin to value empathy for its utility rather than its authenticity, confusing comfort with conscience.

## Affective overload: the saturation of feeling

A related consequence of the compassion illusion is affective overload—a state of emotional saturation that arises when constant empathic signaling from digital systems overwhelms rather than enriches human emotional life (Caglar-Ozhan et al., 2022; Hazer-Rau et al., 2020). In such environments, users experience a persistent low-level engagement with simulated empathy, leading to emotional fatigue and diminished sensitivity to genuine affective cues. Affective layer is now present in every interaction, from recommendation algorithms to wellness trackers: “You seem stressed,” “Let's take a deep breath,” “I'm here for you.” These signals, while perceived as supportive, lead to a situation where the user is continuously engaged at a low level with emotional feedback (Hazer-Rau et al., 2020).

Psychologically, this creates an environment of perpetual affective stimulation. Individuals receive empathy-like responses not just when distressed but in ordinary daily interactions. Over time, genuine emotional experience becomes entangled with algorithmic reinforcement, producing emotional inflation—a gradual dulling of sensitivity to authentic empathy. When compassion becomes ubiquitous, it risks losing meaning (Caglar-Ozhan et al., 2022).

Affective overload also has cognitive costs. The brain's emotion-regulation systems depend on cycles of activation and rest. Constant exposure to simulated compassion can interrupt these cycles, leading to fatigue and decreased capacity for deep empathy (Gustafsson and Hemberg, 2022; Henkel et al., 2020). Thus, the very technologies designed to support emotional wellbeing may paradoxically produce emotional exhaustion.

## Reclaiming authentic empathy

Recognizing the compassion illusion does not entail rejecting emotional AI. When used intentionally, such technologies can expand access to care, enhance self-reflection, and support mental health. The challenge lies in distinguishing augmentation from replacement. Machines can assist emotional awareness but should not become its primary medium.

To preserve authenticity, several interventions are necessary:

1. Design for transparency: Emotional AI systems should clearly signal the fact that they are artificial. The use of false anthropomorphism—implicating the presence of consciousness or feeling—should be completely avoided. It is only fair that the users are informed that empathy is being simulated, not really felt.
2. Embed reflective friction: AI could, instead of giving seamless responses, include reflective pauses or prompts that would coax the users into processing their feelings independently and that AI would be there to support them. For instance, after a supportive response, a chatbot could give a suggestion such as: “Do you want to talk about this with a friend or a counselor?” Such friction is still promoting human reconnection, though.
3. Cultivate emotional literacy: The discussions on algorithmic empathy should be held in the education system and mental health programs as it will help the individuals to recognize its strengths and limits. That is, awareness can prevent dependence on it and strengthen human empathy as a different skill.
4. Reassert moral agency: Compassion should be considered an ethical act and not just a means of communication. It has to be ensured through policies and professional ethics that affective technologies promote rather than secretly manipulate human beings' good qualities.

Ultimately, genuine empathy is not measured by accuracy but by presence—the capacity to remain with another's suffering without control or agenda. While artificial systems may simulate affective expression, they lack the conscious intentionality required for moral engagement. Preserving human empathy therefore demands resisting the appeal of emotionally convenient, automated forms of care.

## Conclusion

The emergence of artificial empathy marks a significant development in how emotional experience is mediated through technology. For the first time, people are engaging with systems that communicate in the language of care without possessing consciousness or compassion. This encounter demonstrates both technological progress and the enduring human desire for emotional connection. Our willingness to accept simulation as recognition reveals as much about our social needs as it does about machine design.

The challenge, however, is not simply emotional but ethical and systemic. Empathy that lacks lived experience is reflective rather than relational; it reproduces emotion without vulnerability and care without accountability. If left unchecked, such imitation risks eroding the moral basis of interaction, replacing genuine reciprocity with algorithmic responsiveness.

Future work in affective computing and human–AI interaction must therefore address not only how machines simulate empathy but why and to what extent they should. This calls for the design of transparent affective systems that disclose their artificiality, promote reflective user engagement, and support rather than substitute human empathy. Regulatory frameworks and ethical standards should evolve to ensure that artificial empathy serves therapeutic, educational, and accessibility goals without manipulating emotion or moral judgment.

AI can thus help illuminate the contours of our emotional life, but it cannot inhabit them. To remain ethically and psychologically grounded, we must cultivate empathy as a human capacity rather than an engineered performance. The task ahead is not to make machines feel, but to design systems that preserve the integrity of feeling itself.
